# Comprehensive signature analysis of drug metabolism differences in the White, Black and Asian prostate cancer patients

**DOI:** 10.18632/aging.203158

**Published:** 2021-06-18

**Authors:** Yang Liu, Jia-Wei Zhou, Cun-Dong Liu, Jian-Kun Yang, De-Ying Liao, Zhi-Jian Liang, Xiao Xie, Qi-Zhao Zhou, Kang-Yi Xue, Wen-Bing Guo, Ming Xia, Jun-Hao Zhou, Ji-Ming Bao, Cheng Yang, Hai-Feng Duan, Hong-Yi Wang, Zhi-Peng Huang, Shan-Chao Zhao, Ming-Kun Chen

**Affiliations:** 1 Department of Urology, The Third Affiliated Hospital of Southern Medical University, Guangzhou 510630, China; 2 Department of Urology, Nanfang Hospital, Southern Medical University, Guangzhou 510515, China

**Keywords:** prostate cancer, race, drug resistance, drug metabolism, comprehensive signature

## Abstract

The drug response sensitivity and related prognosis of prostate cancer varied from races, while the original mechanism remains rarely understood. In this study, the comprehensive signature including transcriptomics, epigenome and single nucleotide polymorphisms (SNPs) of 485 PCa cases- including 415 Whites, 58 Blacks and 12 Asians from the TCGA database were analyzed to investigate the drug metabolism differences between races. We found that Blacks and Whites had a more prominent drug metabolism, cytotoxic therapy resistance, and endocrine therapy resistance than Asians, while Whites were more prominent in drug metabolism, cytotoxic therapy resistance and endocrine therapy resistance than Blacks. Subsequently, the targeted regulation analysis indicated that the racial differences in cytotoxic therapy resistance, endocrine therapy resistance, might originate from drug metabolisms, and 19 drug metabolism-related core genes were confirmed in the multi-omics network for subsequent analysis. Furthermore, we verified that *CYP1A1, CYP3A4, CYP2B6, UGT2B17, UGT2B7, UGT1A8, UGT2B11, GAS5, SNHG6, XIST* significantly affected antineoplastic drugs sensitivities in PCa cell lines, and these genes also showed good predictive efficiency of drug response and treatment outcomes for PCa in this cohort of patients. These findings revealed a comprehensive signature of drug metabolism differences for the Whites, Blacks and Asians, and it may provide some evidence for making individualized treatment strategies.

## INTRODUCTION

Prostate cancer is the second most common cancer, and the fifth leading cause of death from cancer in men worldwide [1, 2]. However, the incidence and mortality of PCa were varied significantly from different races [3, 4]. African Americans were reported to have the highest incidence worldwide, while the White and Black people are at a higher risk than Asian people for suffering from PCa [2, 5]. Moreover, the White and Black people have poorer cancer specific survival (CSS) and overall survival (OS) for PCa than Asian people [6–8]. Meanwhile, the incidence of prostate cancer has been in a rising trend amongst all races in recent years [9, 10]. However, the original mechanism for these differences among the races has still not been fully understood until recently.

It is reported that dietary patterns and geographical environment differences may be the explanation for PCa incidence differences among ethnic groups [11–13]. However, the reasons for different treatment outcomes amongst the ethnicities are still not entirely clear. There was a study that reported that racial differences in genetic variants have an impact on drug sensitivities, clinical signs of progress and treatment options for prostate cancer [14]. In addition, Bernard B. et al reported that Black, White, and Asian people have differences in their responses to chemotherapy and endocrine therapy, which lead to different survival benefits [15]. These results suggest that drug response and treatment outcomes for PCa differ between racial and ethnic groups. However, systematic comparisons of race differences in drug treatments (cytotoxic therapy, endocrine therapy, molecular targeting therapy, and so on) have not been reported yet, and the mechanisms of the treatment response differentiations amongst ethnicities are still not well understood.

What drives racial differences in drug sensitivity and treatment outcomes of PCa patients, and are there any genetic differences amongst races that lead to the differences in drug metabolic capacity? In recent years, sequencing technology has provided new methods that allow researchers to easily review hundreds of tumor profiles and discover the genetic alterations responsible for drug metabolism. In our study, we aimed to investigate the drug metabolism differences in the White, Black and Asian ethnicities, using a comprehensive signature involving transcriptomics, epigenome and SNPs, so as to systematically compare the significant differences in drug metabolism-related pathways, drug sensitivities among ethnicities, and to account for the racial differences in treatment outcomes. Finding drug metabolism-related core genes in multi-omics and predicting treatment outcomes for PCa, these findings may provide effective and novel evidence for the personalized treatment of PCa.

## RESULTS

### Multi-omics genetic signatures differences in White, Black and Asian PCa patients

**Figure 1:**
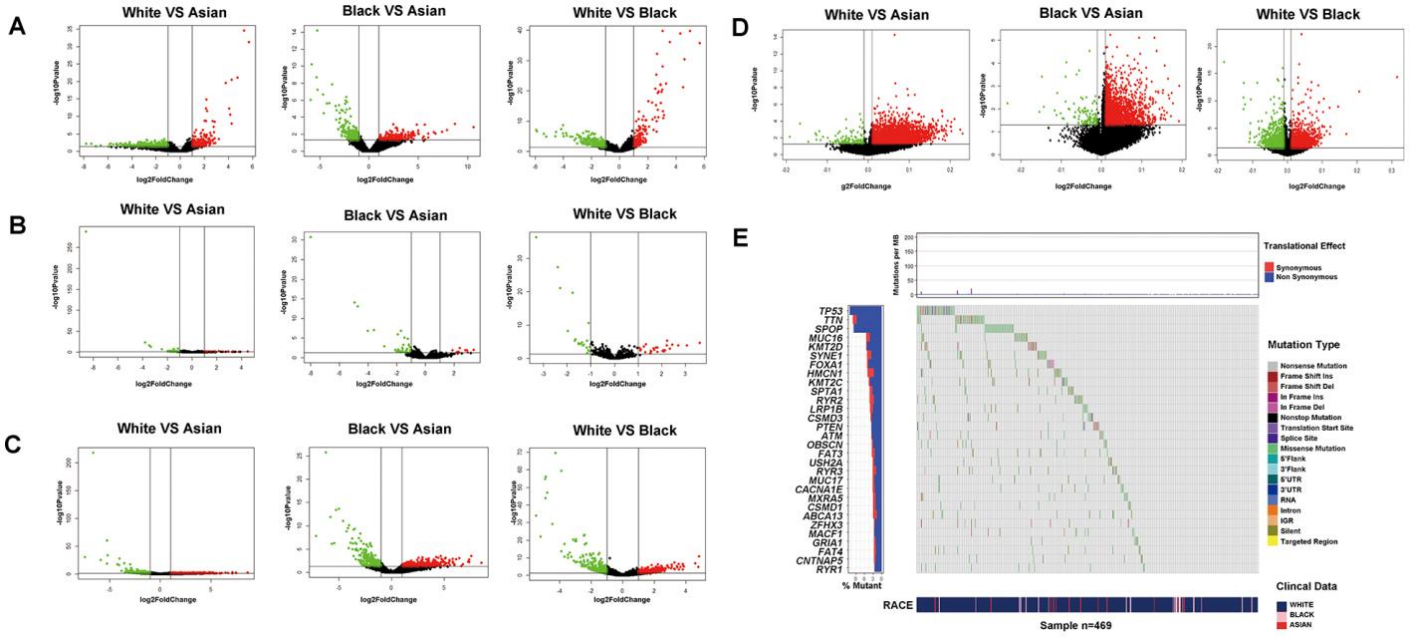
**Transcriptomics, epigenome and SNPs difference analysis for PCa of different RACES.** mRNA expression (**A**) miRNA expression (**B**) lncRNA expression (**C**) DNA methylation level (**D**) differential analysis for White people and Asian people, Black people and Asian people, White people and Black people, respectively. (**E**) SNPs status differential analysis (such as mutation rate, mutation type.ect) for White, Black and Asian people in TCGA patients.

Firstly, we compared the differences of transcriptomics, epigenome and SNPs among White, Black and Asian people. From a total of 19676 official mRNA gene symbols, 470 differentially expressed genes (DEGs) (including 253 up-regulated genes and 217 down-regulated genes) were identified when comparing White people to Asian people, 396 DEGs were identified (including 204 up-regulated genes and 192 down-regulated genes) when comparing Black people to Asian people, 483 DEGs were identified (including 307 up-regulated genes and 176 down-regulated genes) when comparing White people to Black people, respectively (Figure 1A). From a total of 1881 official miRNA gene symbols, 51 DEGs were identified (including 14 up-regulated genes and 37 down-regulated genes) when comparing White people to Asian people, 50 DEGs were identified (including 10 up-regulated genes and 40 down-regulated genes) when comparing Black people to Asian people, 45 DEGs were identified (including 25 up-regulated genes and 20 down-regulated genes) when comparing White people to Black people, respectively (Figure 1B). From a total of 14447 official lncRNA gene symbols, 600 DEGs were identified (including 340 up-regulated genes and 260 down-regulated genes) when comparing White people to Asian people, 618 DEGs were identified (including 354 up-regulated genes and 264 down-regulated genes) when comparing Black people to Asian people, 648 DEGs were identified (including 317 up-regulated genes and 331 down-regulated genes) when comparing White people to Black people, respectively (Figure 1C). From a total of 29004 official methylation gene symbols, 3567 differential methylation genes were identified (including 3437 up-regulated genes and 130 down-regulated genes) when comparing White people to Asian people, 2285 differential methylation genes were identified (including 2174 up-regulated genes and 111 down-regulated genes) when comparing Black people to Asian people, 3659 differential methylation genes were identified (including 1916 up-regulated genes and 1743 down-regulated genes) when comparing White people to Black people, respectively (Figure 1D). From a total of 11202 official SNP gene symbols, 10697 mutations were noted in White people, 1679 mutations in Black people, 320 mutations in Asian people- of which White people had more prominent mutations of *TP53, ATM*; Black people had more prominent mutations in *ATM, TP53, CDK12* than Asian people; White people had more prominent mutations in TP53 than Black people (Figure 1E and Supplementary Figure 1). Surprisingly, we found that the mutations of *TP53, ATM, CDK12* showed significant resistance to chemotherapy of pan-cancer cell lines in the GDSC database. For example, *TP53* mutation showed significant resistance to paclitaxel, 5-Fluorouracil, doxorubicin and gemcitabine. The details are summarized in Supplementary Tables 1, 2.

### Drug metabolism differences in White, Black and Asian PCa patients

**Figure 2:**
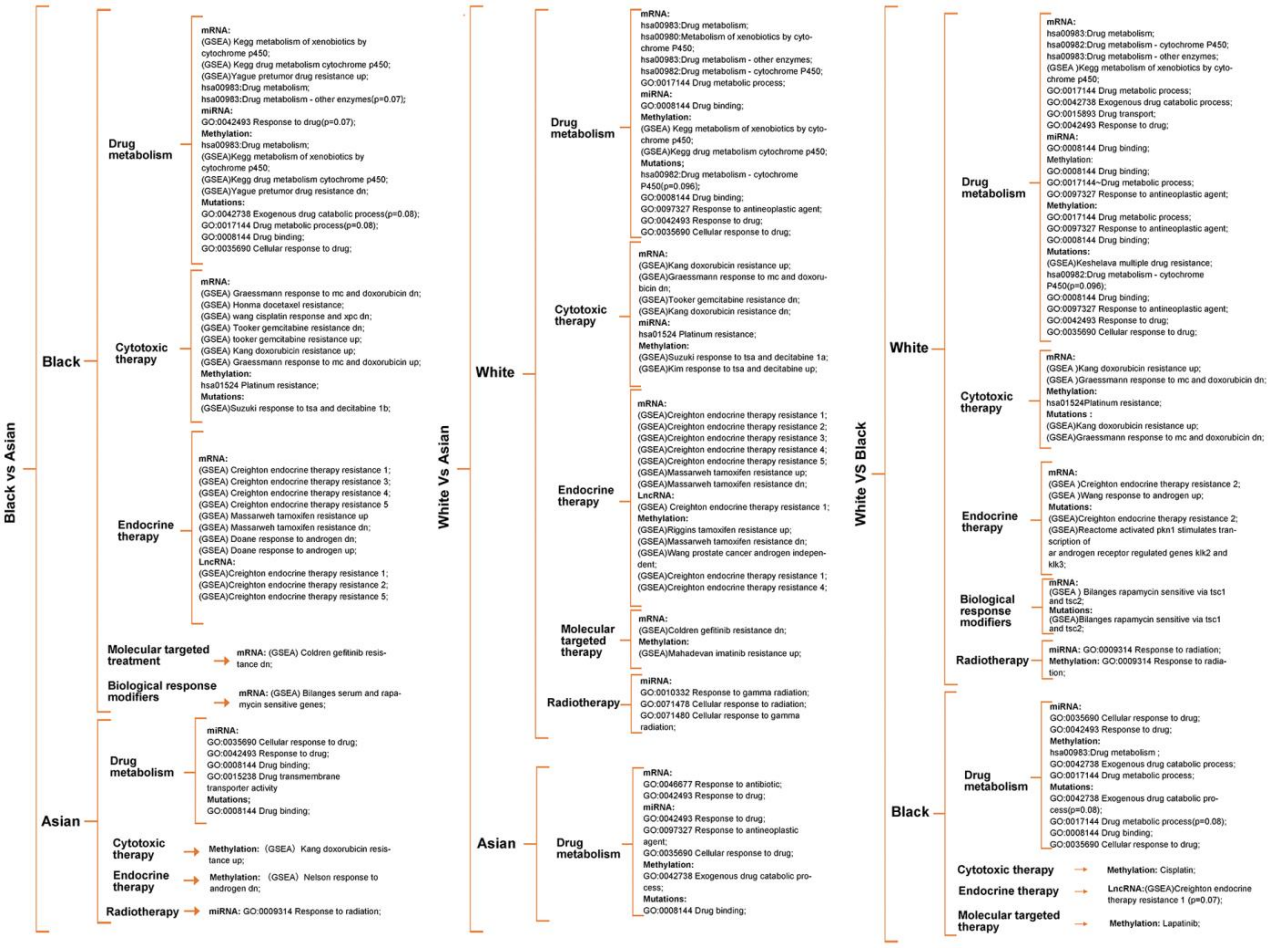
**Metabolism pathway difference analysis according to multi-omics for races, of which drug metabolism, cytotoxic therapy, endocrine therapy, radiotherapy, molecular targeted therapy, biological response modifiers therapy differences were the main focus.** Unless otherwise specified, all the significance P value < 0.05.

Enrichment analysis was carried out according to the differential genes in transcriptomics, epigenome and SNPs, to identify the differences in metabolism pathway for White, Black and Asian PCa patients. We found significant differences in drug metabolism, cytotoxic therapy, endocrine therapy, molecular targeting treatment, biological response modifiers and radiotherapy amongst the ethnicities. More precisely, Black people were more prominently enriched in DMP (hsa00982: drug metabolism - cytochrome P450, hsa00980: metabolism of xenobiotics by cytochrome P450, hsa00983:drug metabolism), GSEA: pretumor drug resistance, GSEA: docetaxel resistance, GSEA: doxorubicin resistance, GSEA: gemcitabine resistance, GSEA: gefitinib resistance, GSEA: endocrine therapy resistance, GSEA: response to androgens, GSEA: serum and rapamycin sensitive genes in mRNA level, GSEA: endocrine therapy resistance in lncRNA level, DMP, hsa01524:platinum drug resistance, GSEA: pretumor drug resistance in methylation level when compared with Asian people; White people were more prominently enriched in DMP, hsa00983: drug metabolism - other enzymes, GO:0017144 drug metabolic process, GSEA: doxorubicin resistance, GSEA: gemcitabine resistance, GSEA: gefitinib resistance, GSEA: endocrine therapy resistance in mRNA level, hsa01524:platinum resistance, GO:0010332 response to gamma radiation in miRNA level, GSEA: endocrine therapy resistance in lncRNA level, hsa00980: metabolism of xenobiotics by cytochrome P450, hsa01524: platinum drug resistance, GSEA: endocrine therapy resistance, GSEA: response to androgens pathways in methylation level when compared with Asian people; White people were more prominently enriched in DMP, hsa00983: drug metabolism - other enzymes, GO:0017144 drug metabolic process, GO:0042738 exogenous drug catabolic process, GSEA: doxorubicin resistance, GSEA: endocrine therapy resistance, GSEA: response to androgen, GSEA: rapamycin sensitive via tsc1 and tsc2 in mRNA level, GO:0009314 response to radiation pathway in miRNA level, GO:0017144 drug metabolic process, hsa01524:platinum resistance, GO:0009314 response to radiation in methylation level when compared with Black people. (Figure 2, and Supplementary Figure 4, Supplementary Tables 3–6).

It was worth noting that Black people were also prominently enriched in hsa00983: drug metabolism, GO:0042738 exogenous drug catabolic process in methylation level when compared with White people (Figure 2), that is because parts of methylation level have an uncertain relationship with gene expression. Therefore, we screened methylation drivers genes for each race for further enrichment analysis (Supplementary Figure 2 and Supplementary Table 8), and the results verified that the Black people were more prominently enriched in hsa00982 drug metabolism, GO:0017144 drug metabolic process, GO:0009314 response to radiation when compared with the Asian people; White people were more prominently enriched in hsa00982 drug metabolism, GO:0017144 drug metabolic process, GO:0009314 response to radiation when compared with Asian people; White people were more prominently enriched in molecular targeting treatment when compared with Black people (Supplementary Figure 3 and Supplementary Table 6); Further enrichment analysis of mutated genes of the different races showed that SNPs might relate to hsa00982:drug metabolism - cytochrome P450 (p=0.096), GSEA: multiple drug resistance, GO:0097327 response to antineoplastic agent, GSEA: doxorubicin resistance, GSEA: endocrine therapy resistance, GSEA: response to androgen pathways in White people, GO:0042738 exogenous drug catabolic process(p=0.08), GO:0017144 drug metabolic process(p=0.08), GSEA: response to tsa and decitabine 1b pathways in Black people, GO:0008144 drug binding pathway in Asian people, respectively (Figure 2 and Supplementary Table 7). Unless otherwise specified, all significant P value was < 0.05.

### Key functional modules with ethnic differences were identified for each omics

**Figure 3:**
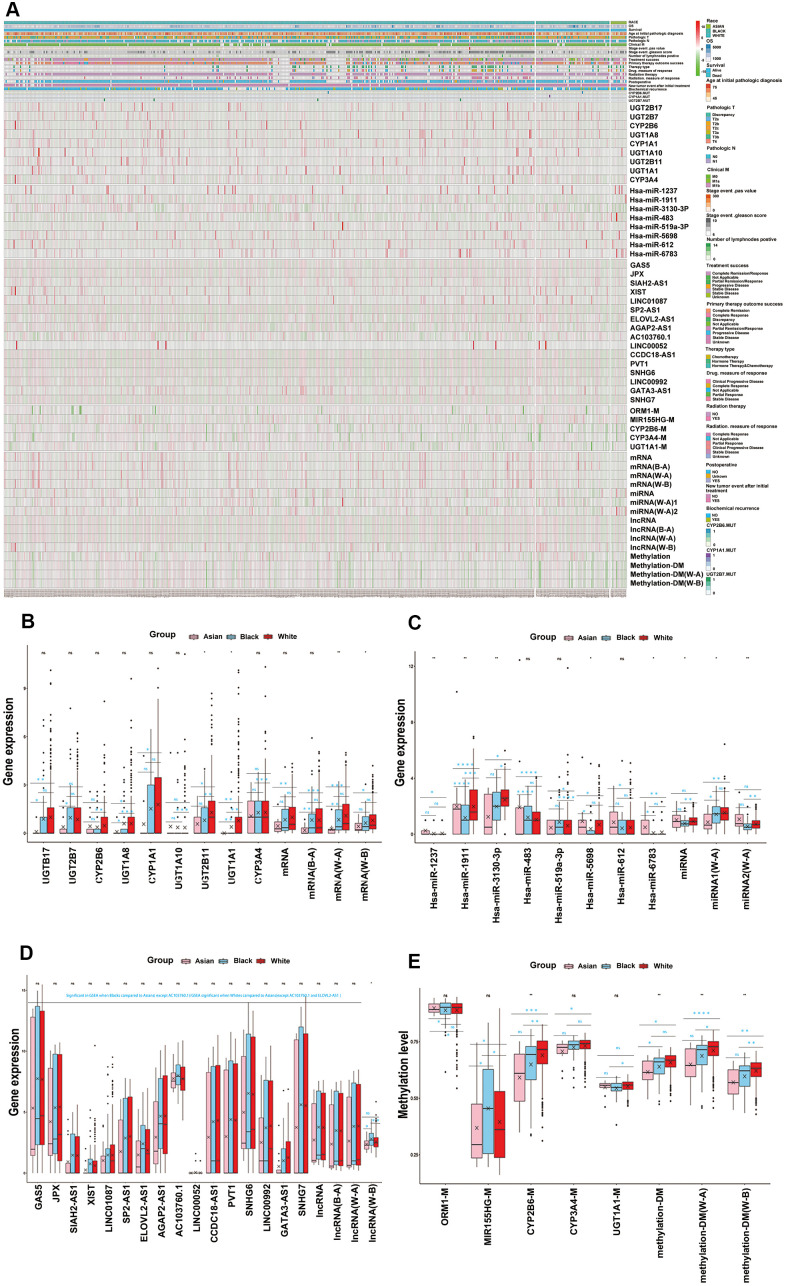
**Key functional modules with ethnic differences were identified for each omics.** (**A**) Multi-omics key functional modules core genes differences analysis for races, as both single gene or total, which were shown in hot map. (**B**) The core genes of mRNA key functional module (drug metabolism) differences analysis for RACES as both single gene or total. (**C**) The core genes of miRNA key functional module (platinum resistance and antineoplastic agent response) differences analysis for RACES as both single gene or total. (**D**) The core genes of lncRNA key functional module (endocrine therapy resistance) differences analysis for RACES as both single gene or total. (**E**) The core genes of methylation key functional modules (drug metabolism, molecular targeted therapy and response to radiation) differences analysis for RACES as both single gene or total. (Notes: mRNA, miRNA, lncRNA, methylation expressed as the mean value of the core genes of each key functional module, methylation-DM expressed as the mean value of drug mentalism-related methylations, miRNA 1 expressed as the mean value of antineoplastic agent response related core miRNAs, miRNA 2 expressed as the mean value of platinum resistance related core miRNAs, B-A, W-A, W-B expressed as the mean value of core genes of each key functional modules which significant in Black people VS Asian people, White people VS Asian people, White people VS Black people, respectively).

We summarized the important functional modules, including drug metabolism, cytotoxic therapy, endocrine therapy, molecular targeting treatment, biological response modifiers, radiotherapy, and related genes set amongst the different races (Supplementary Table 9). We found that the differences of these important functional modules amongst the different ethnicities were caused by the combination of transcriptomics, epigenome and SNPs. Furthermore, we found that each omics had its own prominent functional module. We defined these prominent functional module as the key functional module for each omics, more precisely, drug metabolism, platinum resistance and antineoplastic agent response, endocrine therapy resistance, molecular targeted therapy, and response to radiation were identified as the key functional modules for mRNA, miRNA, lncRNA, DNA methylation, respectively. It is worth noting that DNA methylation and SNPs also significantly occurred in drug metabolism modules (Table 1 and Supplementary Table 9). What’s more, we preliminarily identified the core genes or target genes in multi-omics key functional modules, and the DNA methylation and SNP genes, which occurred in the drug metabolism functional modules, were also included as the preliminary core genes. After screening, we affirmed 9 core mRNA which were related to drug metabolism, 8 core miRNA which were related to platinum resistance and antineoplastic agent response, 16 core lncRNA which were related to endocrine therapy resistance, 1 core methylation for molecular targeted therapy and 1 core methylation for response to radiation. In addition, 3 methylation and 3 SNPs in the drug metabolism functional module were also included in the core genes of DNA methylation and SNPs. (Table1 and Supplementary Figure 5A–5C). In addition, multi-omics key functional modules core genes showed significant differences amongst White, Black and Asian people, as both single gene or total, the details of which are shown in Table 1 and Figure 3.

**Table 1:** Comparison of the multi-omics key functional modules and related core genes among RACES.

**Functional modules**	**Omics**	**Genes**	**Comparison**	**Expression significant**	**Functional significant**
Drug metabolism	mRNA	UGT2B17	Asian VS Black	Black	Black
	mRNA	UGT1A8	Asian VS Black	Black	Black
	mRNA	UGT2B7	Asian VS Black	Black	Black
	mRNA	UGT1A1	Black VS White	White	White
	mRNA	CYP3A4	Black VS White	White	White
	mRNA	UGT1A10	Black VS White	White	White
	mRNA	UGT1A8	Black VS White	White	White
	mRNA	CYP2B6	Black VS White	White	White
	mRNA	UGT2B11	Black VS White	White	White
	mRNA	UGT1A1	Asian VS White	White	White
	mRNA	UGT2B17	Asian VS White	White	White
	mRNA	UGT1A8	Asian VS White	White	White
	mRNA	CYP1A1	Asian VS White	White	White
	mRNA	UGT2B11	Asian VS White	White	White
	Methylation	CYP2B6	White VS Asian	White	White
	Methylation	CYP3A4	White VS Asian	White	White
	Methylation	CYP2B6	White VS Black	White	White
	Methylation	UGT1A1	White VS Black	White	White
	Mutations	UGT2B7	**——**	White	White
	Mutations	CYP2B6	**——**	Black	Black
	Mutations	CYP1A1	**——**	Black	Black
Platinum drug resistance	miRNA	hsa-miR-1911-3p	Asian VS White	Asian	White
	miRNA	hsa-miR-612	Asian VS White	Asian	White
	miRNA	hsa-miR-1237-3p	Asian VS White	Asian	White
	miRNA	hsa-miR-5698	Asian VS White	Asian	White
	miRNA	hsa-miR-483-3p	Asian VS White	Asian	White
	miRNA	hsa-miR-6783-3p	Asian VS White	Asian	White
Response to antineoplastic agent	miRNA	hsa-miR-3130-3p	Asian VS White	White	Asian
	miRNA	hsa-miR-519a-3p	Asian VS White	White	Asian
Endocrine therapy resistance	lncRNA	LINC00052	Black VS Asian	Black	Black
	lncRNA	LINC01087	Black VS Asian	Black	Black
	lncRNA	JPX	Black VS Asian	Black	Black
	lncRNA	CCDC18-AS1	Black VS Asian	Black	Black
	lncRNA	PVT1	Black VS Asian	Black	Black
	lncRNA	SIAH2-AS1	Black VS Asian	Black	Black
	lncRNA	SNHG7	Black VS Asian	Black	Black
	lncRNA	LINC00992	Black VS Asian	Black	Black
	lncRNA	SNHG6	Black VS Asian	Black	Black
	lncRNA	GATA3-AS1	Black VS Asian	Black	Black
	lncRNA	SP2-AS1	Black VS Asian	Black	Black
	lncRNA	GAS5	Black VS Asian	Black	Black
	lncRNA	ELOVL2-AS1	Black VS Asian	Black	Black
	lncRNA	XIST	Black VS Asian	Black	Black
	lncRNA	AGAP2-AS1	Black VS Asian	Black	Black
	lncRNA	LINC01087	White VS Asian	White	White
	lncRNA	JPX	White VS Asian	White	White
	lncRNA	PVT1	White VS Asian	White	White
	lncRNA	SIAH2-AS1	White VS Asian	White	White
	lncRNA	CCDC18-AS1	White VS Asian	White	White
	lncRNA	SNHG7	White VS Asian	White	White
	lncRNA	SNHG6	White VS Asian	White	White
	lncRNA	LINC00992	White VS Asian	White	White
	lncRNA	GAS5	White VS Asian	White	White
	lncRNA	XIST	White VS Asian	White	White
	lncRNA	SP2-AS1	White VS Asian	White	White
	lncRNA	GATA3-AS1	White VS Asian	White	White
	lncRNA	AGAP2-AS1	White VS Asian	White	White
	lncRNA	ELOVL2-AS1	White VS Black	White	White
	lncRNA	LINC00052	White VS Black	White	White
	lncRNA	AC103760.1	White VS Black	White	White
	lncRNA	XIST	White VS Black	White	White
Molecular targeted therapy	Methylation	ORM1	White VS Asian	Asian	White
	Methylation	ORM1	Black VS Asian	Asian	Black
Response to radiation	Methylation	MIR155HG	White VS Asian	Asian	White
	Methylation	MIR155HG	Black VS Asian	Asian	Black

### Drug resistance differences amongst races might originate from drug metabolism

**Figure 4:**
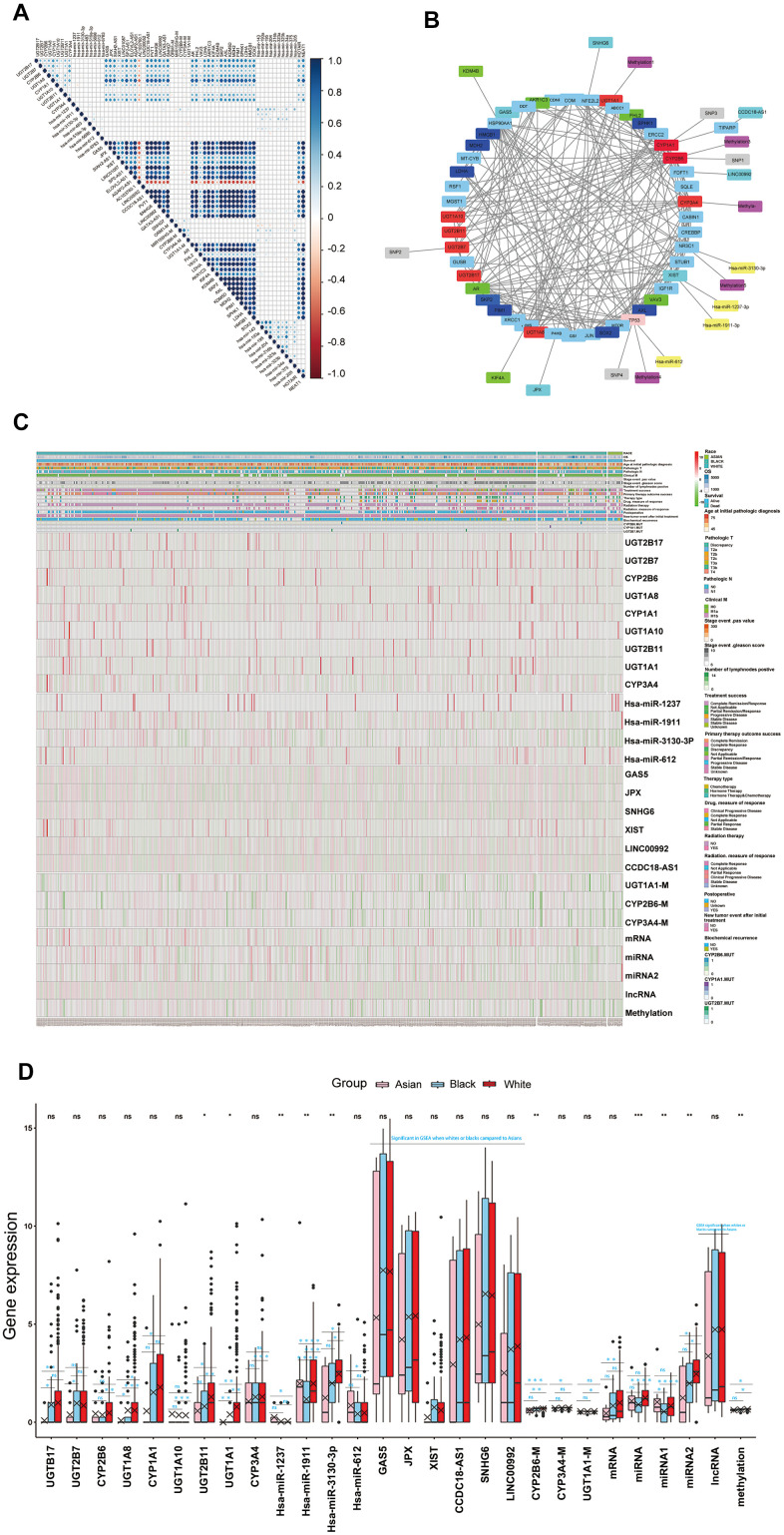
(**A**) The correlations of the core genes of each key functional modules to each other and the correlations of the core genes of each key functional modules to cytotoxic resistance genes (*HMGB1*, docetaxel resistance: *SKP2, AXL, KDM5D, MDH2, PIM1, SPHK1, LDHA, SOX2, Hsa-mir-143, Hsa-mir-193a, Hsa-mir-195a, Hsa-mir-204, Hsa-mir-216b, Hsa-mir-323a, Hsa-323b, Hsa-mir-34a, Hsa-mir-375*, platinum resistance: *Hsa-mir-205, HOTAIR, NEAT1*), endocrine therapy resistance genes (*AR, FHL2, VAV3, LDHA, AKR1C3, KIF4A, KDM4B*) reported the in literature. (**B**) The core genes or targets of each key functional modules in this work and cytotoxic resistance genes (*HMGB1*, docetaxel resistance: *SKP2, AXL, MDH2, PIM1, SPHK1, LDHA, SOX2*), endocrine therapy resistance genes (*AR, FHL2, VAV3, LDHA, AKR1C3, KIF4A, KDM4B*) reported in the literature, were taken for regulatory network analysis, the genes which regulated well from each other in network were identified as drug metabolism-related core genes for further study. (**C**, **D**) Drug metabolism-related core genes differences analysis for RACES as both single gene or total, which were shown in the hot map and box plots. (Notes: mRNA, miRNA, lncRNA, methylation expressed as the mean value of the core genes of each key functional modules in network, miRNA 1 expressed as the mean value of antineoplastic agent response related core miRNAs in network, miRNA 2 expressed as the mean value of platinum resistance related core miRNAs in network).

Firstly, correlation analysis was adopted for multi-omics key functional modules that indicated that the endocrine therapy resistance functional module had a strong positive correlation with the drug metabolism module in this work. Meanwhile, the cytotoxic resistance genes (*HMGB1*, docetaxel resistance: *SKP2, AXL, KDM5D, MDH2, PIM1, SPHK1, LDHA, SOX2, Hsa-mir-143, Hsa-mir-193a, Hsa-mir-195a, Hsa-mir-204, Hsa-mir-216b, Hsa-mir-323a, Hsa-323b, Hsa-mir-34a, Hsa-mir-375,* platinum resistance: *Hsa-mir-205, HOTAIR, NEAT1*), endocrine therapy resistance genes (*AR, FHL2, VAV3, LDHA, AKR1C3, KIF4A, KDM4B*) reported in the literature also showed strong positive correlations with the drug metabolism functional module. Furthermore, the platinum resistance functional module showed positive correlations with cytotoxic treatment resistance genes reported in the literature, endocrine therapy resistance functional module showed positive correlations with endocrine therapy resistance genes reported in the literature (Figure 4A). These evidences suggested that the cytotoxic treatment resistance and endocrine therapy resistance showed good correlations to drug metabolism in PCa. Next, multi-omics key functional modules core genes or targets in this work, and cytotoxic resistance genes (*HMGB1*, docetaxel resistance: *SKP2, AXL, MDH2, PIM1, SPHK1, LDHA, SOX2*), endocrine therapy resistance genes (*AR, FHL2, VAV3, LDHA, AKR1C3, KIF4A, KDM4B*) reported in literature were taken for regulatory network analysis, which demonstrated that drug metabolism module, cytotoxic treatment resistance module, and endocrine therapy resistance module were well regulated with each other (Figure 4B, and Supplementary Figure 5F, Supplementary Table 10). Therefore, we have reason to think that the differences in cytotoxic treatment resistance, endocrine therapy resistance amongst the different races might have originated from drug metabolism (Figure 4A, 4B and Supplementary Figure 5D–5F). Regulatory network analysis identified that mRNA (*UGT2B17, UGT1A8, UGT2B7, UGT1A1, CYP3A4, UGT1A10, CYP2B6, UGT2B11, CYP1A1*), miRNA (*hsa-miR-1237-3p, hsa-miR-1911-3p, hsa-miR-3130-3p, hsa-miR-612*), lncRNA (*XIST, CCDC18-AS1, GAS5, JPX, SNHG6, LINC00992*), SNPs (*UGT2B7, CYP2B6, CYP1A1*), methylation (*UGT1A1, CYP3A4, CYP2B6*) were well regulated to each other, and to cytotoxic resistance genes or endocrine therapy resistance genes reported in literature, and these genes were deemed as drug metabolism-related core genes in further studies (Supplementary Table 11). We also found that drug metabolism-related core genes showed differences in transcriptomics, epigenome and SNPs, as both single gene or total, amongst the different races, which lead to drug metabolism, and drug resistance differences amongst the White, Black and Asian patients. (Figure 4C, 4D and Table 1).

### Drug metabolism-related core genes affected drug sensitivities in PCa cell lines

Multi-platform data was used to verify the correlations between multi-omics drug metabolism-related core genes and drug sensitivities of those antineoplastic compounds in prostate cancer cell lines. In other words, we want to further confirm the changes of IC50, EC50 or AUCs when targeting drug metabolism-related core genes in PCa treatment. Combined with CCLE, GDSC and CTRP database, results showed that mRNA (*CYP1A1, CYP3A4, UGT1A1, UGT1A8, UGT1A10, UGT2B7, UGT2B11, UGT2B17*), miRNA (*hsa-miR-612*), lncRNA (*GAS5, JPX, XIST, CCDC18-AS1*), methylation (*CYP1A1, CYP2B6,* GAS5*,* SNH6) affected the IC50, EC50 and AUCs of antineoplastic drugs in prostate cancer cell lines. It was encouraging that the expression of *CYP3A4* and *GAS5,* methylation levels of CYP1A1 had strong correlations to paclitaxel resistance, and *UGT2B7* had strong correlations to doxorubicin resistance in PCa treatment. In addition, we unexpectedly found that the methylation SNH6 was closely related to paclitaxel resistance in PCa treatment. These details are summarized in Table 2. These genes are expected to be effective targets for the treatment of prostate cancer. Furthermore, we found that *CYP1A1, CYP2B6, CYP3A4, UGT1A8, UGT2B11, UGT2B17, UGT2B7, XIST, SNHG6* were also well affected the chemotherapy or endocrine therapy drug sensitivities (which define as (1−AUC) in GDSC and define as [1−(AUC/30)] in CTRP) of antineoplastic drugs in pan-cancer cell lines according to GDSC and CTRP database; For example, *CYP1A1, CYP2B6, CYP3A4, UGT2B11, UGT2B17, UGT2B7, XIST* showed resistance to docetaxel, *CYP1A1, CYP2B6, CYP3A4, UGT2B11, UGT2B17, XIST* showed resistance to cisplatin, *CYP2B6, UGT1A8, UGT2B11, XIST, SNHG6* showed resistance to abiraterone, and so on. We will not enumerate them all here, but the details are summarized in Supplementary Table 12. Considering the above results, we have taken *CYP1A1, CYP2B6, CYP3A4, UGT1A8, UGT2B11, UGT2B17, UGT2B7, GAS5, XIST, SNHG6,* which might be more relevant to chemotherapy or endocrine therapy resistance in PCa treatment, for subsequent study.

**Table 2:** The correlations between multi-omics drug metabolism-related core genes to IC50, EC50 or AUCs of antineoplastic drugs in CCLE/GDSC/CTRP database for PCa cell lines.

**Omics**	**Platform**	**Genes**	**Effect**	**Compounds**	**Correlations**	**Pvalue**
mRNA	CCLE	CYP1A1	IC50	Panobinostat	-0.999	0.024
mRNA	CCLE	CYP1A1	IC50	Paclitaxel	-0.997	0.049
mRNA	CCLE	CYP3A4	IC50	Panobinostat	0.999	0.033
mRNA	CCLE	CYP3A4	IC50	Paclitaxel	1	0.008
mRNA	CCLE	UGT1A8	IC50	Sorafenib	-1	0.001
mRNA	CCLE	UGT1A8	IC50	AZD0530	0.999	0.033
mRNA	CCLE	UGT1A8	IC50	Lapatinib	-1	0.001
mRNA	CCLE	UGT2B17	IC50	Sorafenib	-0.999	0.026
mRNA	CCLE	UGT2B17	IC50	AZD0530	1	0.008
mRNA	CCLE	UGT2B17	IC50	Lapatinib	-0.997	0.026
mRNA	CCLE	UGT2B11	IC50	Sorafenib	-1	0.004
mRNA	CCLE	UGT2B11	IC50	AZD0530	0.999	0.034
mRNA	CCLE	UGT2B11	IC50	Lapatinib	-1	<0.001
miRNA	CCLE	hsa-miR-612	IC50	Crizotinib	1	0.019
miRNA	CCLE	hsa-miR-612	IC50	AEW541	0.999	0.033
lncRNA	CCLE	GAS5	IC50	Paclitaxel	0.997	0.047
lncRNA	CCLE	JPX	IC50	RAF265	-1	0.004
methylation	CCLE	GAS5	IC50	Erlotinib	-1	<0.001
methylation	CCLE	GAS5	IC50	Paclitaxel	-0.999	0.021
methylation	CCLE	GAS5	IC50	Panobinostat	-0.997	0.046
methylation	CCLE	SNH6	IC50	Paclitaxel	0.999	0.021
methylation	CCLE	SNH6	IC50	Panobinostat	-0.997	0.049
methylation	CCLE	CYP1A1	IC50	PF2341066	1	<0.001
methylation	CCLE	CYP1A1	IC50	AEW541	1	0.014
mRNA	CCLE	UGT1A10	EC50	17-AAG	-0.998	0.041
mRNA	CCLE	UGT1A10	EC50	PLX4720	1	0.017
lncRNA	CCLE	XIST	EC50	17-AAG	-0.998	0.036
lncRNA	CCLE	CCDC18-AS1	EC50	17-AAG	-1	0.012
lncRNA	CCLE	CCDC18-AS1	EC50	PLX4720	1	0.012
methylation	CCLE	GAS5	EC50	Lapatinib	-1	0.007
methylation	CCLE	GAS5	EC50	PF2341066	1	0.017
methylation	CCLE	GAS5	EC50	TKI258	-0.999	0.020
methylation	CCLE	GAS5	EC50	RAF265	-1	0.017
methylation	CCLE	SNH6	EC50	Lapatinib	0.999	0.028
methylation	CCLE	SNH6	EC50	PF2341066	-0.998	0.038
methylation	CCLE	SNH6	EC50	Panobinostat	0.999	0.034
methylation	CCLE	SNH6	EC50	RAF265	1	0.004
methylation	CCLE	SNH6	EC50	TKI258	0.998	0.042
methylation	CCLE	CYP1A1	EC50	Topotecan	1	0.002
methylation	CCLE	CYP1A1	EC50	Paclitaxel	1	0.013
methylation	CCLE	CYP2B6	EC50	PLX4720	0.998	0.041
mRNA	GDSC	UGT2B11	IC50	PLX4720	-1	0.011
mRNA	GDSC	UGT2B7	AUCs	Doxorubicin	1	0.014
mRNA	GDSC	UGT2B17	AUCs	Nutlin-3a	-1	0.011
lncRNA	GDSC	XIST	AUCs	PLX4720	1	0.020
mRNA	CTRP	CYP1A1	AUCs	Lapatinib	-1	0.012
mRNA	CTRP	CYP3A4	AUCs	Lapatinib	0.997	0.046
mRNA	CTRP	CYP3A4	AUCs	Gefitinib	0.997	0.049
mRNA	CTRP	UGT2B7	AUCs	Sorafenib	1	0.002
mRNA	CTRP	UGT1A1	AUCs	Nutlin-3a	-0.999	0.022
mRNA	CTRP	hsa-miR-612	AUCs	Nutlin-3a	1	0.012
lncRNA	CTRP	JPX	AUCs	Erlotinib	-0.998	0.041
lncRNA	CTRP	GAS5	AUCs	Gefitinib	-1	0.009
methylation	CTRP	GAS5	AUCs	Gefitinib	-0.997	0.049
methylation	CTRP	GAS5	AUCs	Lapatinib	-0.997	0.046
methylation	CTRP	SNH6	AUCs	Lapatinib	0.998	0.036
methylation	CTRP	CYP1A1	AUCs	Tamoxifen	0.998	0.041
methylation	CTRP	CYP1A1	AUCs	Nutlin-3a	0.999	0.031

IC50: half-maximal inhibitory concentration. EC50: half-maximal effect concentration. AUCs: Area under the dose–response curve.

### Drug metabolism-related core genes predict drug response and treatment outcomes for PCa patients

**Figure 5:**
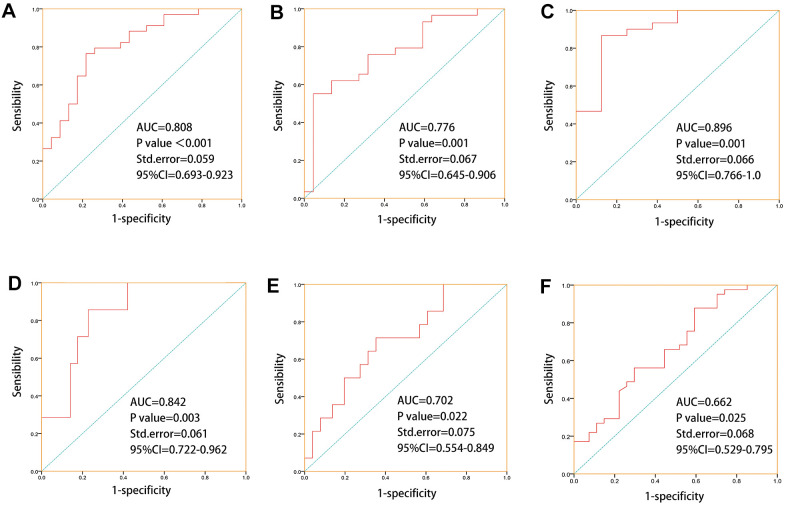
**ROC prediction models were established for “primary therapy outcome success, treatment success, drug response, stage event.** PSA value, biochemical recurrence, new tumor event after initial treatment” based on drug metabolism-related core genes which with good correlations to chemotherapy or endocrine therapy drugs sensitivities. (**A**) ROC prediction model for primary treatment outcome success (AUC=0.808, P=0, SD=0.059). (**B**) ROC prediction model for treatment success (AUC =0.776, P=0.001, SD=0.067). (**C**) ROC prediction model for drug response (AUC =0.896, P=0.001, SD=0.066). (**D**) ROC prediction model for stage event. PSA value (AUC =0.842, P=0.003, SD=0.061). (**E**) ROC prediction model for biochemical recurrence (AUC =0.702, P=0.022, SD=0.075). (**F**) ROC prediction model for new tumor event after initial treatment(AUC =0.662, P=0.025, SD=0.068).

Later, we combined *CYP1A1, CYP2B6, CYP3A4, UGT1A8, UGT2B11, UGT2B17, UGT2B7, GAS5, XIST, SNHG6,* which showed good correlations to chemotherapy or endocrine therapy resistance, to predict drug response and treatment outcomes in 69 PCa patients who have previously received chemotherapy or endocrine therapy. The result of ROC prediction model showed that these genes have a good predictive power of primary treatment outcome success (AUC =0.808, P<0.001, SD=0.059), treatment success (AUC =0.776, P=0.001, SD=0.067), drug response(AUC =0.896, P=0.001, SD=0.066), stage event PSA value (AUC =0.842, P=0.003, SD=0.061), biochemical recurrence (AUC =0.702, P=0.022, SD=0.075), new tumor event after initial treatment (AUC =0.662, P=0.025, SD=0.068), for TCGA PCa patients, after receiving chemotherapy or hormone therapy (Figure 5). These models demonstrate that these genes were potential biomarkers for predicting the drug response and treatment outcomes of prostate cancer. These genes also showed significant racial differences- specifically, *CYP1A1, CYP3A4, UGT1A8, UGT2B11, UGT2B17, GAS5, XIST, SNHG6* were more significant in White people in comparison to Asian people, *UGT1A8, UGT2B17, UGT2B7, GAS5, XIST, SNHG6* were more significant in Black people in comparison to Asian people, and *CYP2B6, UGT1A8, UGT2B11, CYP3A4* were more significant in White people in comparison to Black people (Supplementary Figure 6).

## DISCUSSION

Several studies have shown racial differences in the genetic background of prostate cancer patients. For example, Brandon A Mahal. et al have revealed racial differences in the mutational profiles of 2393 prostate cancer patients, which including 2109 White, 204 Black, and 80 Asian [16], Timothy R Rebbeck. et al have also proposed that individuals of European/Asian ancestry have different risk alleles than individuals of African ancestry in PCa patients [17]. In our study, we provide a more complete and novel finding of racial differences in prostate cancer among White, Black, and Asian men, especially for drug treatment and drug metabolism, based on transcriptome, epigenome and SNPs, and these results may increase the contributions to this field.

Recently, NATURE published the latest results based on whole-genome, whole-transcriptome and DNA methylation data, revealing the genomic changes in Chinese patients were markedly different from those in Western patients (41% *FOXA1* mutation in PCa as the most prominent signature in the Chinese population), and emphasized the importance of individualized treatment based on ethnic genetic background [18]. Meanwhile, Mahal BA. et al reported that FOXA1 has the highest mutation frequency in the Asian population when compared with Blacks and Whites in both primary and metastatic prostate cancer patients [16]. These findings further supported our results. We know that FOXA1 has been reported to help shape AR signaling and drives growth and survival of prostate cancer cells [19], Which may be a potential explanation for the differences in prognosis among White, Black, and Asian PCa patients. What’s more, it was reported that ATM, PTEN in metastatic prostate cancer had a higher mutation frequency in Blacks and Whites than in Asians, and TP53, CDK12 in primary prostate cancer had a higher mutation frequency in Whites and Asians than in Blacks [16]. Most of these results are in line with ours, we also found different mutations in ATM, TP53 and CDK12 among White, Black, and Asian PCa populations. It’s reported that TP53, PTEN, ATM and CDK12 were more mutated in metastatic castration-resistant prostate cancer (mCRPC) [20]. And we found that TP53, ATM and CDK12 mutations showed significant resistance to chemotherapy of pan-cancer cell lines. Therefore, the differences in gene mutations may be related to drug sensitivity and prognosis among different ethnic groups.

Several studies have shown that overall survival (OS) of the Black populations is shorter than the White populations in PCa, but the Black OS was almost equal with the White OS after the docetaxel treatment [21], which might be due to the racial differences in drug sensitivities. There were studies that claimed that the differences in survival rates between Black and White people might due to selection bias or a possible biological difference between PCa. In addition, there may be ethnic differences in pharmacological and pharmacokinetic criteria that may affect the performance of therapeutic drugs such as docetaxel. In addition, many genes, including *KDM5D*, have been shown to modulate docetaxel sensitivity in prostate cancer [22–23]. In our study, many new genes were also found to modulate docetaxel sensitivity, such as CYP1A1, CYP3A4, GAS5, SHN6, etc, and race differences in the expression modulation of these genes may be a potential explanation for these observations. What’s more, we found that these genes contribute well to drug metabolic pathways and differ significantly among ethnic groups, and ethnic differences in resistance to cytotoxic therapy and endocrine therapy might result from the differences in drug metabolism.

Recent research claimed that treatments of prostate cancer reflect unexpected ethnic disparities [24]. Moses et al. showed that African American (AA) men were less likely than Whites to receive treatment for radical prostatectomy, external beam radiation therapy, or brachytherapy [25]. This preference may influence prognosis or outcome for each race. Moreover, a few studies have compared the response effects of chemotherapy or endocrine therapy for prostate cancer of different races, but no consistent and systematic conclusions have been drawn [15]. To this end, we systematically compared the differences of White, Black and Asian people in chemotherapy, endocrine therapy, radiotherapy and molecular targeted therapy. et al, and further found that the differences in drug resistance might originate from drug metabolism among different races.

In order to provide an effective target for the personalized treatment of prostate cancer, we identified drug metabolism-related core genes which related to drug metabolism, drug sensitivities, and related treatment outcomes in multi-omics, such as *CYP1A1, CYP2B6, CYP3A4, UGT1A8, UGT2B11, UGT2B17, UGT2B7, GAS5, XIST, SNHG6*. et al. Several studies have shown that *GAS5, SNH6, UGT1A10, UGT2B17* were well related to clinical prognosis, *CYP3A4* related to paclitaxel resistance and therapeutic effects of abiraterone and enzalutamide in PCa [26–32]. Which supported our results, and these findings based on the multi-omics genetic structure of races could better guide the individualized treatment for PCa.

In conclusion, transcriptomics, epigenome and SNPs were significant differences in Whites, Blacks and Asians of PCa, which directly lead to the differences in drug metabolism, drug resistance pathways. What’s more, drug metabolism promoted drug resistance differences among races, which lead to the differences in drug responses and treatment outcomes of PCa. Therefore, these findings can help us to understand the mechanisms of the difference in drug metabolism among prostate cancer patients in different races and help us in making individualized treatment strategies.

## MATERIALS AND METHODS

### Data sources

In our study, 485 PCa samples including 415 White people(85.6%), 58 Black people (12.0%) and 12 Asian people (2.5%) were acquired from the TCGA data portal (https://tcga-data.nci.nih.gov/tcga/), of which transcriptomics, epigenome, SNPs data and corresponding clinical information were included. After being matched with clinical data, 479 samples with mRNA count (including 411 White, 56 Black and 12 Asian people), 477 samples with microRNA count (including 407 White, 58 Black and 12 Asian people), 476 samples with lncRNA count (including 408 White, 56 Black and 12 Asian people), 469 samples with SNPs (including 402 White, 55 Black and 12 Asian people), 483 samples with DNA methylation FPKM (including 414 White, 57 Black and 12 Asian people) were contained. In these patients, 69 samples (14.2%) have received hormone therapy or chemotherapy (Figure 4A). Meanwhile, transcriptomics, epigenome, SNPs, half-maximal inhibitory concentration (IC50), and half-maximal effect concentration (EC50) of 24 antineoplastic compounds for 22RV1, DU145 and PC3 were downloads from the CCLE database (https://portals.broadinstitute.org/ccle/data). Transcriptomics, epigenome, SNPs and the area under the dose response curve (AUCs) of 24 antineoplastic compounds for pan-cancer cell lines were acquired from the CTRP database (http://portals.broadinstitute.org/ctrp/?page=#ctd2BodyHome). Transcriptome, the half-maximal inhibitory concentration (IC50), and the area under the dose-response curve (AUCs) of 22 antineoplastic compounds for pan-cancer cell lines were acquired from the GDSC database (https://www.cancerrxgene.org/gdsc1000/GDSC1000_WebResources//Home.html). Authorization was not requested from a local ethics committee, as all data were available on an open-access platform.

### Multi-omics difference analysis

Differentially expressed genes of mRNAs, microRNAs, and lncRNAs were identified by the edge R package and differentially expressed DNA methylation genes were identified by the limma package for all races. The setting cutoffs for upregulated and downregulated genes were included in the fold change feature |logFC| > 1 for mRNAs, microRNA and lncRNAs, and |logFC| > 0.01 for DNA methylation, with a significant P value of < 0.05.

### Identification of DNA methylation driven genes

The R package MethylMix was applied to identify methylation-driven genes, which are defined as differential DNA methylation genes that negatively correlation to gene expression.

### Target gene prediction analysis

Target genes were predicted for miRNA and lncRNA, the targets of miRNA were identified by Mirwalk (http://mirwalk.umm.uni-heidelberg.de/), and the targets of lncRNA were identified by Encori (http://starbase.sysu.edu.cn/index.php).

### Enrichment analysis

Enrichment analysis was used to investigate differential functions or signaling pathways for the different races, which was completed by GSEA, DAVID and webgestalt together to increase the credibility of the results. mRNA, lncRNA, DNA methylation, and SNPs were enriched by GSEA (http://www.broadinstitute.org/gsea). mRNA, miRNA target genes, DNA methylation and SNPs were enriched by DAVID (https://david.ncifcrf.gov/summary.jsp) and webgestalt (http://www.webgestalt.org/).

### Multi-omics key functional modules and core genes identify

We identified the most significant pathways in each omics as the key functional modules. The results showed that drug metabolism, platinum resistance and antineoplastic agent response, endocrine therapy resistance, molecular targeted therapy, and response to radiation were the most key functional modules for mRNA, miRNA, lncRNA, and DNA methylation respectively. Next, the STRING database (https://string-db.org) was used to identify the core genes or targets of mRNA and miRNA functional modules. The core genes of lncRNA functional module were identified according to GSEA gene set. Additionally, we also included the methylation and SNPs that occurred in drug metabolism functional module as the core genes for DNA methylation and SNPs.

### Multi-omics key functional modules regulatory network and drug metabolism-related core genes identification

Regulatory network was established according to multi-omics key functional modules related core genes in this work, and cytotoxic resistance genes, endocrine therapy resistance genes reported in the literature. The target relationships of these genes were affirmed by target prediction and protein-protein interaction (PPI) network, and we visualized the target relationships using Cytoscape software. Drug metabolism-related core genes were defined as the multi-omics key functional modules related core genes which regulated well with each other in the network.

### Statistical analysis

Transcriptomics, epigenome data were standardized by log2(x+1) before analysis. Hot map, box plots, volcano map, nomogram and waterfall map and correlation analysis were plotted by R version 3.5.1. Receiver operating characteristic curve(ROC) and all statistical analyses were performed by SPSS 19.0 software (SPSS. Inc., Chicago, IL, USA). In this study, all significant P value was < 0.05.

### Data availability statement

The datasets generated and analyzed during the current study are available in the TCGA, https://portal.gdc.cancer.gov/, CCLE, https://portals.broadinstitute.org/ccle/data, CTRP, http://portals.broadinstitute.org/ctrp/?page=#ctd2BodyHome, and GDSC https://www.cancerrxgene.org/gdsc1000/GDSC1000_WebResources//Home.html.

## Supplementary Material

Supplementary Figures

Supplementary Table 1

Supplementary Table 2

Supplementary Table 3

Supplementary Table 4

Supplementary Table 5

Supplementary Table 6

Supplementary Table 7

Supplementary Table 8

Supplementary Table 9

Supplementary Table 10

Supplementary Table 11

Supplementary Table 12
