# Supplementary material for: Comprehensive signature analysis of drug metabolism differences in the White, Black and Asian prostate cancer patients
Source: Aging (Albany NY). 2021 Jun 18;13(12):16316–40. doi: 10.18632/aging.203158 (PMC8266326; doi:10.18632/aging.203158)
Supplement: Supplementary Figures [file aging-13-203158-s001.pdf]

SUPPLEMENTARY FIGURES

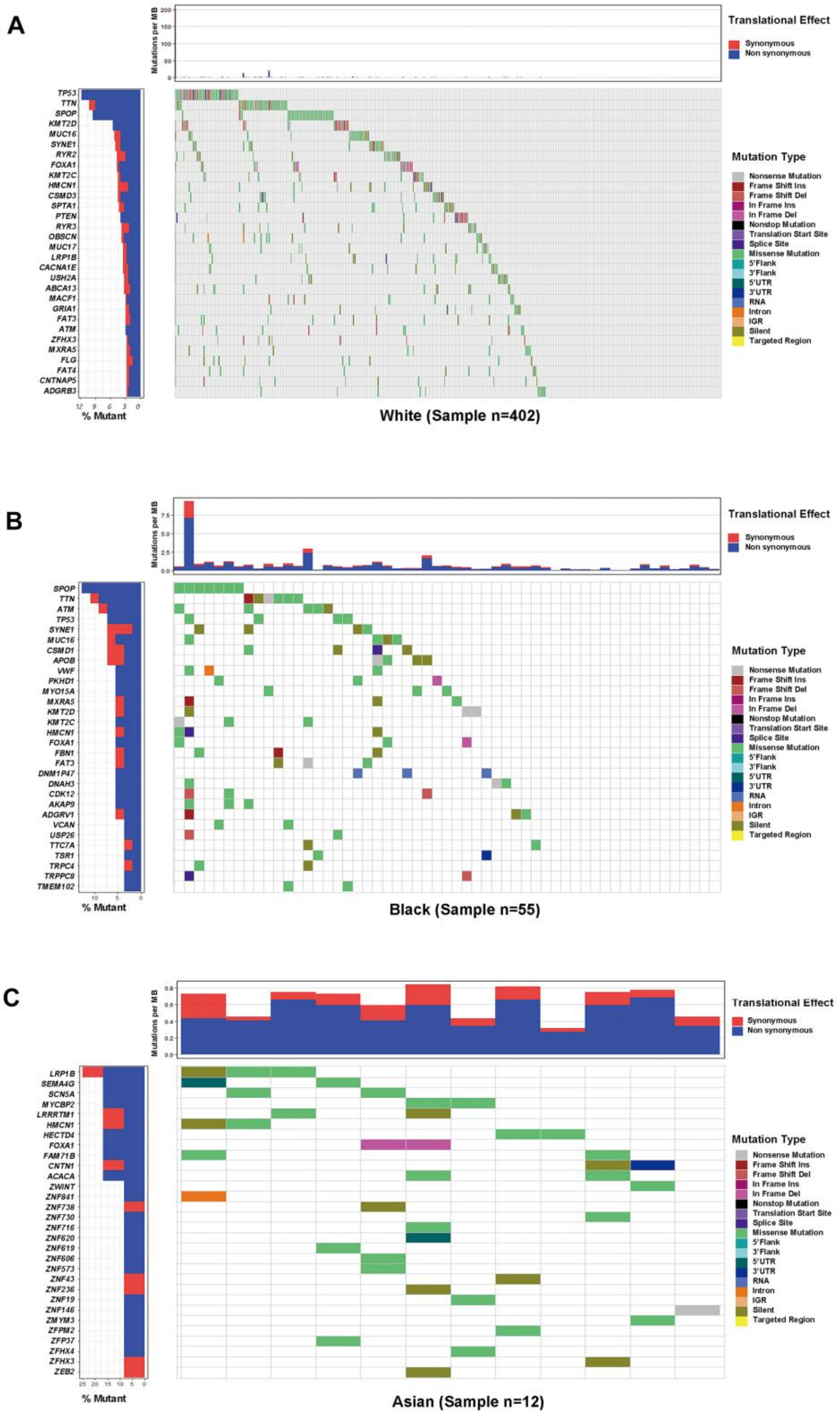

Supplementary Figure 1. SNPs status differential analysis (such as mutation rate, mutation type .ect) for White people (**A**), Black people (**B**) and Asian people (**C**), respectively.

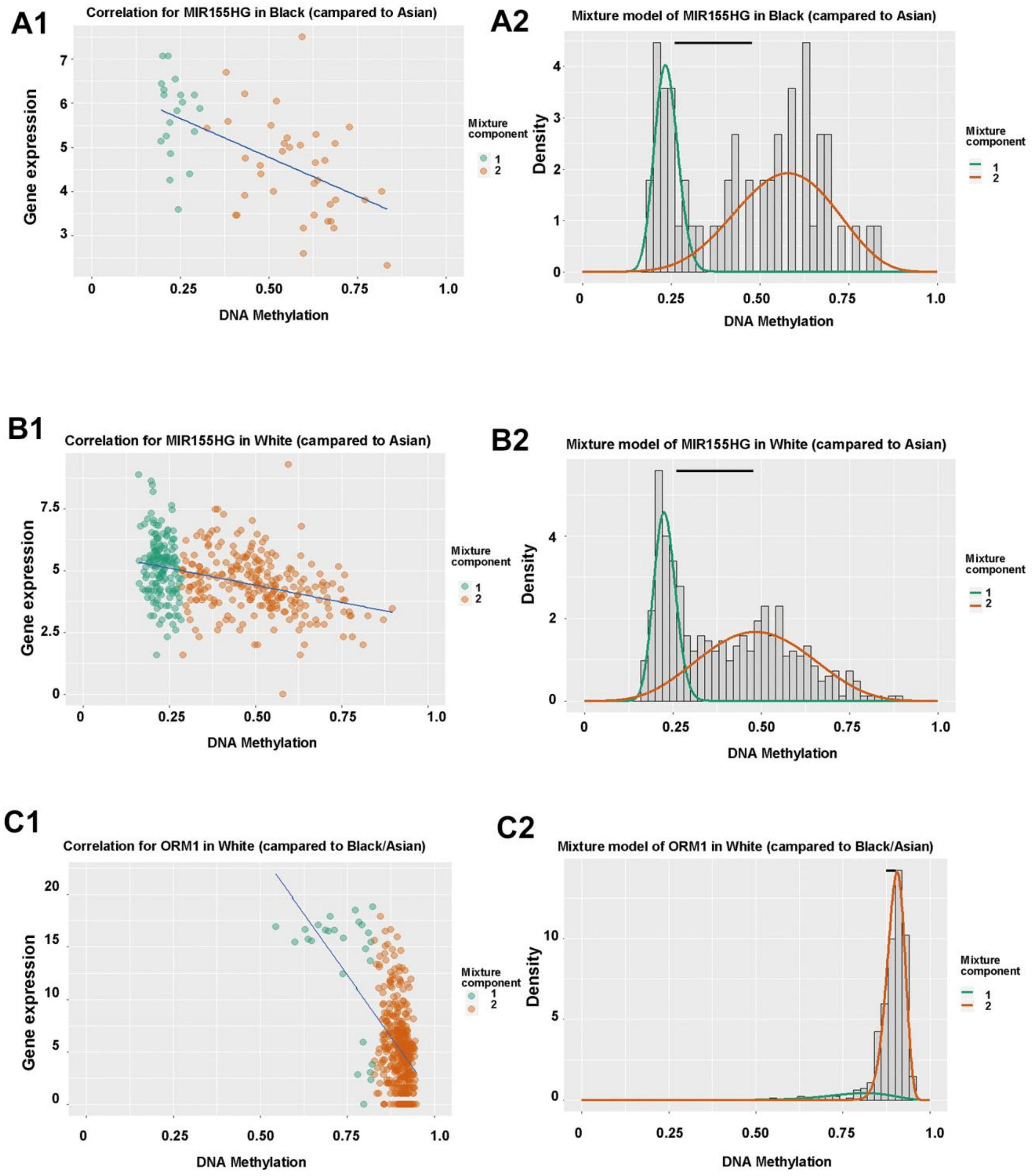

Supplementary Figure 2. Methylation drives genes of prostate cancer identified for RACES (**A**–**C** has shown methylation drivers genes in methylation key functional modules). (**A1**) Regression analysis between the mRNA level and DNA methylation level of *MIR155HG* in Black people; (**A2**) Differential methylation statuses for Black and Asian people. The histogram demonstrates the distribution of *MIR155HG* methylation in Black people. Beta values represent the methylation level (range from 0 to 1), and the horizontal black bar indicates the distribution of methylation values in the Asian people. (**B1**) Regression analysis between the mRNA level and DNA methylation level of *MIR155HG* in White people; (**B2**) Differential methylation statuses for White and Asian people. (**C1**) Regression analysis between the mRNA level and DNA methylation level of *ORM1*in White people; (**C2**) Differential methylation statuses for White and Black people, White and Asian people.

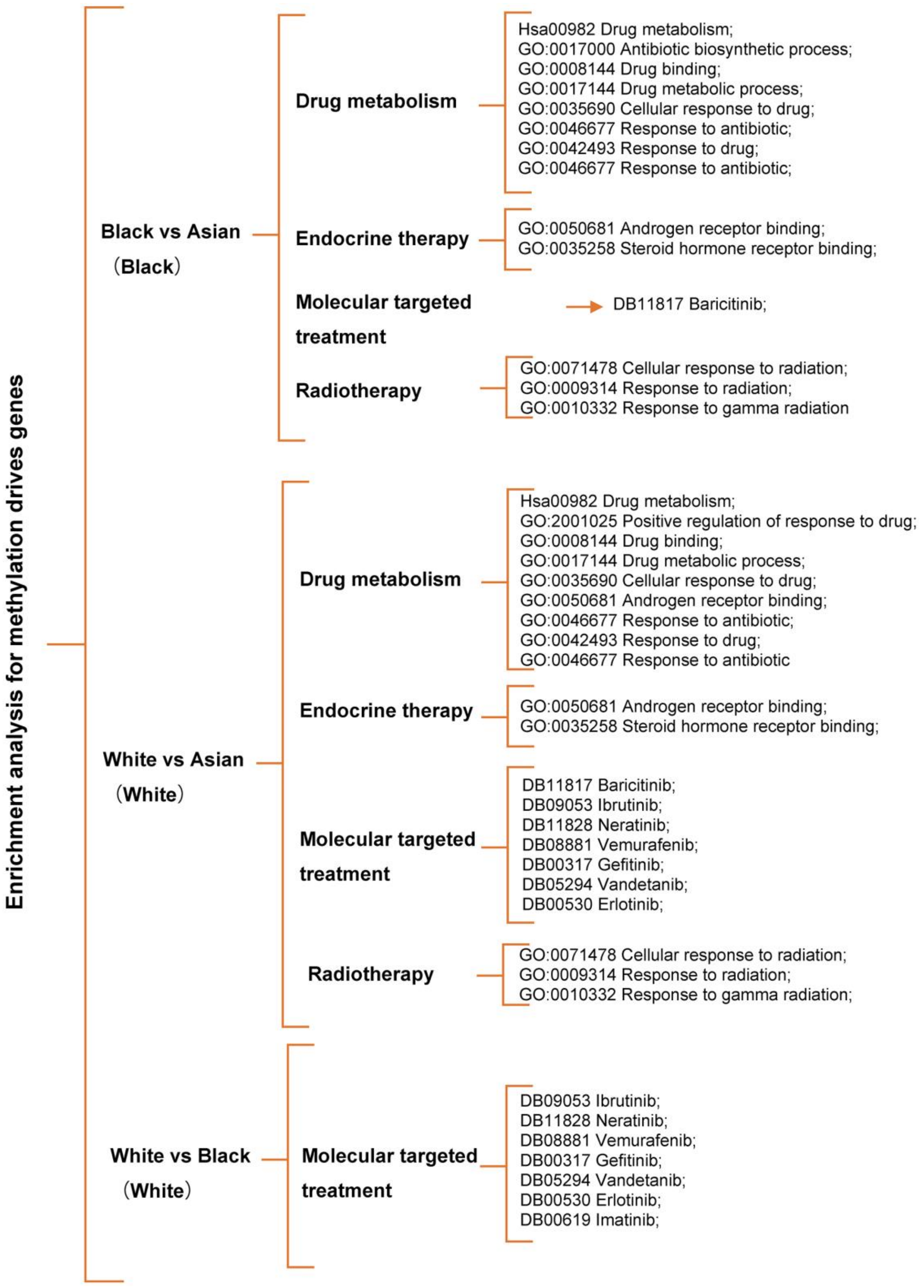

Supplementary Figure 3. Metabolism pathways difference analysis according to methylation drives genes for Black and Asian people, White and Asian people, White and Black people, respectively.

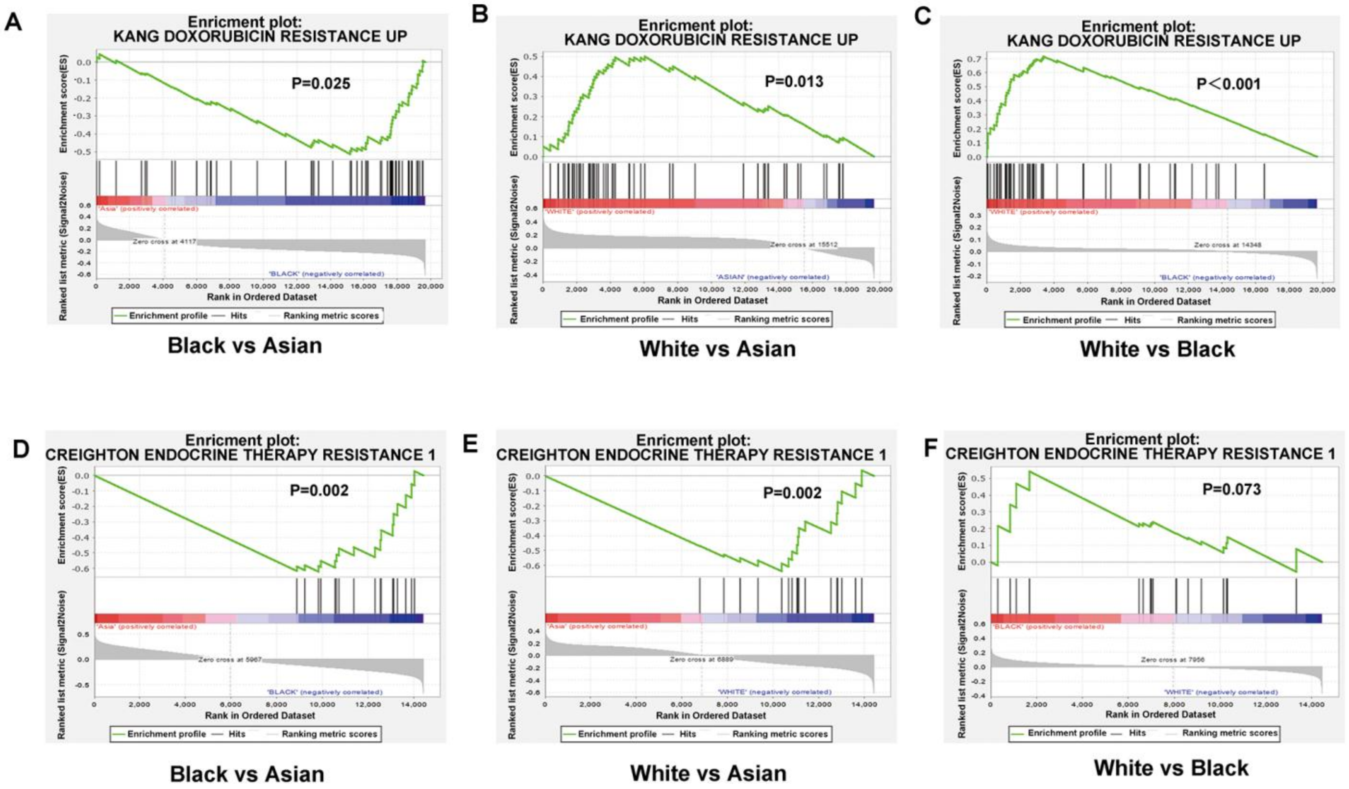

Supplementary Figure 4. GSEA metabolism pathways differences analysis for RACES. (**A**–**C**) Doxorubicin resistance differences analysis based on mRNA for Black and Asian people(p=0.025), White and Asian people (p=0.013), White and Black people(p<0.001), respectively. (**D**–**F**) Endocrine therapy resistance differences analysis based on lncRNA for Black and Asian people(p=0.002), White and Asian people(p=0.002), White and Black people(p=0.073), respectively.

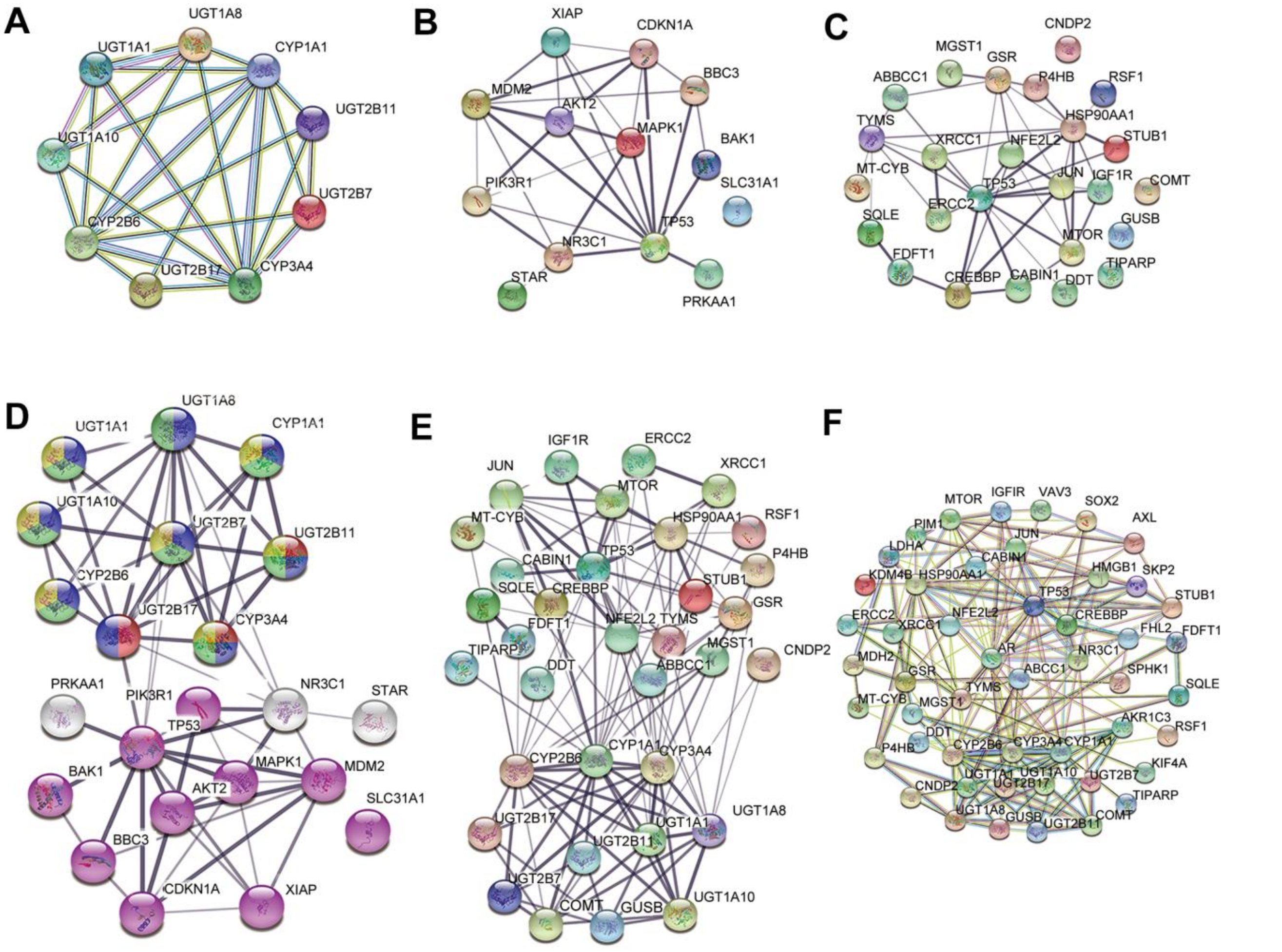

Supplementary Figure 5. Protein-protein interaction (PPI) networks for multi-omics key functional modules. (**A**) PPI network of the core mRNAs of drug metabolism functional module; (**B**) PPI network of the core miRNA target genes of platinum resistance and antineoplastic agent response functional module; (**C**) PPI network of the core lncRNA target genes of endocrine therapy resistance functional module. (**D**) PPI network for the core mRNAs of drug metabolism functional module and the core miRNA target genes of platinum resistance, antineoplastic agent response functional module. (**E**) PPI network for the core mRNAs of drug metabolism functional module and the core lncRNA target genes of endocrine therapy resistance functional module. (**F**) PPI network for the core mRNAs of drug metabolism functional module, the core miRNA target genes of platinum resistance, antineoplastic agent response functional module, the core lncRNA target genes of endocrine therapy resistance functional module and cytotoxic resistance genes (*HMGB1*, docetaxel resistance: *SKP2, AXL, MDH2, PIM1, SPHK1, LDHA, SOX2*), endocrine therapy resistance genes (*AR, FHL2, VAV3, LDHA, AKR1C3, KIF4A, KDM4B*) reported in literature.

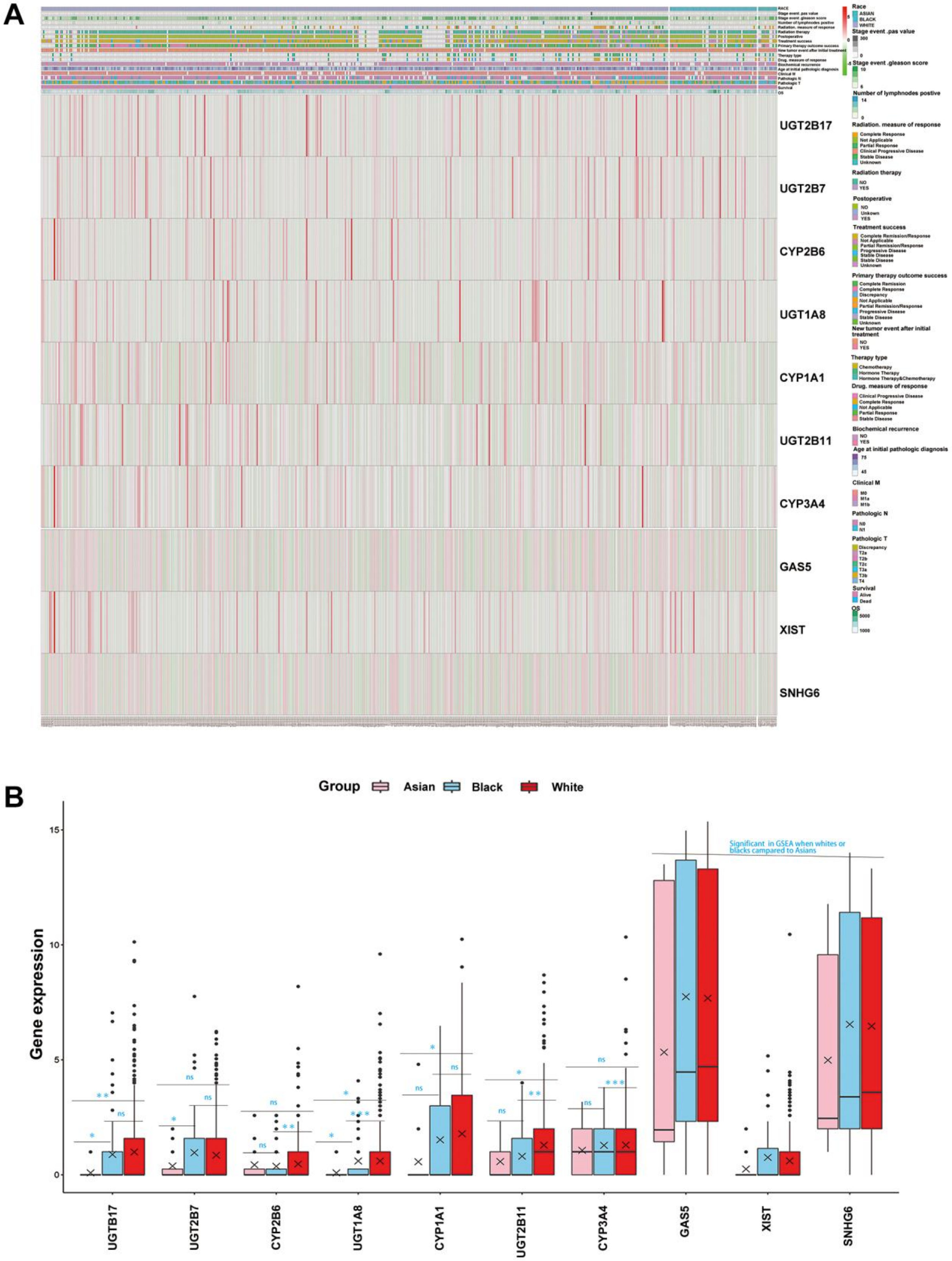

Supplementary Figure 6. The race differences analysis for drug metabolism-related core genes, which more associated with chemotherapy or endocrine therapy resistance in PCa treatment, were shown in the hot map(**A**) and box plots(**B**).
