# Supplementary material for: Comprehensive signature analysis of drug metabolism differences in the White, Black and Asian prostate cancer patients
Source: Aging (Albany NY). 2021 Jun 18;13(12):16316–40. doi: 10.18632/aging.203158 (PMC8266326; doi:10.18632/aging.203158)
Supplement: Supplementary Table 2 [file aging-13-203158-s003.pdf]

Supplementary Table 2. The relationships between mutations and drugs resistance for RACES in the GDSC database.

RACES	FEATURE	Drug name	N_FEATURE _pos	N_FEATURE _neg	FEATUREpos_ logIC50_MEAN	FEATUREneg_ logIC50_MEAN	FEATURE_delta MEAN_IC50	p-value
WHITE/BLACK	ATM_mut	AKT inhibitor VIII	23	855	2.9901	2.2621	0.7281	3.7956E-05
BLACK	CDK12_mut	AR-42	3	42	2.8850	-0.4480	3.3330	8.7294E-06
BLACK	CDK12_mut	PXD101, Belinostat	3	41	2.5424	-0.0143	2.5568	2.1420E-04
WHITE	PTEN_mut	GSK690693	6	40	1.8138	4.4415	-2.6278	3.4885E-06
WHITE	PTEN_mut	GSK690693	89	830	2.5859	3.6637	-1.0777	1.3738E-08
WHITE	PTEN_mut	KU-55933	5	37	2.9444	4.3950	-1.4506	4.5000E-04
WHITE/BLACK	TP53_mut	Dabrafenib	577	293	3.5861	2.6293	0.9568	2.0493E-05
WHITE/BLACK	TP53_mut	CGP-60474	13	13	-1.5947	-2.8274	1.2327	3.0085E-04
WHITE/BLACK	TP53_mut	5-Fluorouracil	12	13	2.3299	0.4615	1.8684	8.3505E-04
WHITE/BLACK	TP53_mut	Mitomycin C	15	4	-1.8361	1.1059	-2.9420	9.9406E-05
WHITE/BLACK	TP53_mut	Bleomycin (50 uM)	616	316	2.5522	1.8817	0.6705	1.7369E-05
WHITE/BLACK	TP53_mut	Doxorubicin	15	4	-2.6881	0.2206	-2.9086	7.1336E-04
WHITE/BLACK	TP53_mut	Doxorubicin	12	13	-0.8158	-2.7925	1.9768	3.4452E-04
WHITE/BLACK	TP53_mut	Gemcitabine	15	4	-3.3618	1.4586	-4.8203	7.2685E-04
WHITE/BLACK	TP53_mut	(5Z)-7-Oxozeaenol	602	311	1.0937	0.3198	0.7740	4.1941E-07
WHITE/BLACK	TP53_mut	Nutlin-3a	12	11	3.6414	1.7152	1.9261	1.6219E-06
WHITE/BLACK	TP53_mut	Nutlin-3a	19	13	3.2950	1.3950	1.9000	7.5961E-08
WHITE/BLACK	TP53_mut	Nutlin-3a	14	29	3.8555	2.1006	1.7549	2.1254E-06
WHITE/BLACK	TP53_mut	Nutlin-3a	23	10	4.3914	2.2671	2.1243	1.3080E-05
WHITE/BLACK	TP53_mut	Nutlin-3a	19	14	4.3994	2.6616	1.7378	1.4118E-04
WHITE/BLACK	TP53_mut	Nutlin-3a	554	292	4.0582	2.5171	1.5411	1.0561E-54
WHITE/BLACK	TP53_mut	Paclitaxel	13	13	-2.1812	-4.4205	2.2393	1.9381E-04
WHITE/BLACK	TP53_mut	EHT 1864	37	4	3.4858	4.8728	-1.3870	5.6949E-05
WHITE/BLACK	TP53_mut	Etoposide	13	13	2.0083	-0.2722	2.2805	7.8310E-04

N:number; pos: positive; neg: negative; FEATUREpos_logIC50_MEAN: Average log IC50 of the postive feature population of pan-cancer cell lines;FEATUREneg_logIC50_MEAN: Average log IC50 of the negative feature population of pan-cancer cell lines;FEATURE_deltaMEAN_IC50: Difference of average natural log IC50 values between the postive and negative feature population of pan-cancer cell lines. In column G, negative value indicates interaction for drug sensitivity, whereas positive value indicates interaction for drug resistance.
