# Supplementary material for: Comprehensive signature analysis of drug metabolism differences in the White, Black and Asian prostate cancer patients
Source: Aging (Albany NY). 2021 Jun 18;13(12):16316–40. doi: 10.18632/aging.203158 (PMC8266326; doi:10.18632/aging.203158)
Supplement: Supplementary Table 8 [file aging-13-203158-s009.pdf]

Supplementary Table 8. Methylation drives genes of prostate cancer for races.

Races	Genes
White (compared to Asian)	GALR1, FABP5, OXGR1, DRC1, CSAG1, HRASLS5, SIAH3, DAPL1, MIR155HG, ACY3, CXCL5, SLURP1, MIA2, RP11-272D20.2, CT83, ADH6, ORM1
Black (compared to Asian)	43535, DDX53, CSAG1, DCAF4L2, RLN1, CLK3P2, WIF1, FBLL1, TMEM26, PEX10, MIR155HG, SNX31, C1orf64, MAOB, MIA2
White (compared to Black)	SNX31, MAGEA3, CLK3P2, DDX53, CSAG1, MAGEC1, RP3-407E4.4, DCAF4L2, DAPL1, C20orf85, MAGEC2, MAGEA6, CALML3, ACY3, CXCL5, C3orf30, CTAG2, CT83, ORM1
