# Supplementary material for: Comprehensive signature analysis of drug metabolism differences in the White, Black and Asian prostate cancer patients
Source: Aging (Albany NY). 2021 Jun 18;13(12):16316–40. doi: 10.18632/aging.203158 (PMC8266326; doi:10.18632/aging.203158)
Supplement: Supplementary Table 11 [file aging-13-203158-s012.pdf]

Supplementary Table 11. Multi-omics drug metabolism-related core genes in regulatory network.

Omics	Core	SNPs	DNA methylation
mRNA	UGT2B17		
mRNA	UGT1A8		
mRNA	UGT2B7	yes	
mRNA	UGT1A1		yes
mRNA	CYP3A4		yes
mRNA	UGT1A10		
mRNA	CYP2B6	yes	yes
mRNA	UGT2B11		
mRNA	CYP1A1	yes	
miRNA	hsa-miR-1237-3p		
miRNA	hsa-miR-1911-3p		
miRNA	hsa-miR-3130-3p		
miRNA	hsa-miR-612		
lncRNA	XIST		
lncRNA	CCDC18-AS1		
lncRNA	GAS5		
lncRNA	JPX		
lncRNA	SNHG6		
lncRNA	LINC00992		
